# The predicted impact of resource provisioning on the epidemiological responses of different parasites

**DOI:** 10.1111/1365-2656.13751

**Published:** 2022-06-14

**Authors:** Diana Erazo, Amy B. Pedersen, Andy Fenton

**Affiliations:** ^1^ Spatial Epidemiology Lab (SpELL) Université Libre de Bruxelles Bruxelles Belgium; ^2^ Institute of Infection, Veterinary & Ecological Sciences University of Liverpool Liverpool UK; ^3^ Institute of Evolutionary Biology School of Biological Sciences University of Edinburgh Edinburgh UK

**Keywords:** *Apodemus sylvaticus*, compartmental models, disease ecology, epidemiology, host–parasite interactions, provisioning, resource levels, supplemental feeding

## Abstract

1. Anthropogenic activities and natural events such as periodic tree masting can alter resource provisioning in the environment, directly affecting animals, and potentially impacting the spread of infectious diseases in wildlife. The impact of these additional resources on infectious diseases can manifest through different pathways, affecting host susceptibility, contact rate and host demography.
2. To date however, empirical research has tended to examine these different pathways in isolation, for example by quantifying the effects of provisioning on host behaviour in the wild or changes in immune responses in controlled laboratory studies. Furthermore, while theory has investigated the interactions between these pathways, this work has focussed on a narrow subset of pathogen types, typically directly transmitted microparasites. Given the diverse ways that provisioning can affect host susceptibility, contact patterns or host demography, we may expect the epidemiological consequences of provisioning to vary among different parasite types, dependent on key aspects of parasite life history, such as the duration of infection and transmission mode.
3. Focusing on an exemplar empirical system, the wood mouse *Apodemus sylvaticus*, and its diverse parasite community, we developed a suite of epidemiological models to compare how resource provisioning alters responses for a range of these parasites that vary in their biology (microparasite and macroparasite), transmission mode (direct, environmental and vector transmitted) and duration of infection (acute, latent and chronic) within the same host population.
4. We show there are common epidemiological responses to host resource provisioning across all parasite types examined. In particular, the epidemiological impact of provisioning could be driven in opposite directions, depending on which host pathways (contact rate, susceptibility or host demography) are most altered by the addition of resources to the environment. Broadly, these responses were qualitatively consistent across all parasite types, emphasising the importance of identifying general trade‐offs between provisioning‐altered parameters.
5. Despite the qualitative consistency in responses to provisioning across parasite types, we predicted notable quantitative differences between parasites, with directly transmitted parasites (those conforming to SIR and SIS frameworks) predicted to show the strongest responses to provisioning among those examined, whereas the vector‐borne parasites showed negligible responses to provisioning. As such, these analyses suggest that different parasites may show different scales of response to the same provisioning scenario, even within the same host population. This highlights the importance of knowing key aspects of host–parasite biology, to understand and predict epidemiological responses to provisioning for any specific host–parasite system.

## Graphical abstract

Given the increasing impact humans are having on resource availability in natural and semi‐natural environments, general theory, ground‐truthed with data from natural host‐parasite systems, can help delevop hypotheses on how different parasite are likely to respond to resource provisioning in natural populations.

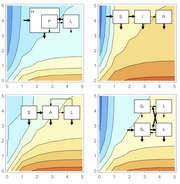

## 1 INTRODUCTION

The availability of food resources is a key environmental factor that impacts the demography and behaviour of animals, but also has the ability to impact host–parasite interactions (Bradley & Altizer, 2007; Robb et al., 2008). Increased resource provisioning, either through natural events such as periodic tree masting, or human activities such as agricultural expansion or the purposeful feeding of wildlife, can alter host–parasite interactions through several distinct pathways acting across multiple ecological scales (Jensen, 1982; Oro et al., 2013). At the individual scale, resource provisioning can improve host body condition and immune responses, which can result in lower susceptibility to infection, shorter‐lasting infections and better health outcomes for the host (Becker et al., 2015; Cypher & Frost, 1999). For example, a study on lace monitors foraging on urban waste suggested that body condition improved as a result of provisioning, and this improvement was associated with a lower intensity of blood‐borne parasites compared to un‐provisioned individuals (Jessop et al., 2012). At the between‐host scale, hosts can aggregate around resources, increasing contact rates and therefore transmission (Boutin, 1990), as seen by the aggregation of European greenfinches around supplemental bird feeders, which increased the transmission of *Trichomonas gallinae* (Lawson et al., 2012). Finally, at the host population scale, parasite prevalence and transmission potential, as measured by the basic reproductive number (*R*_0_), can increase through host demographic responses to provisioning, through the addition of new susceptible individuals to the population via new births or immigration, increases in host carrying capacity or decreases in host mortality rate, all of which can potentially increase the likelihood of parasite persistence (Boutin, 1990; Krebs et al., 1995; Nagy & Holmes, 2005). Therefore, resource provisioning, whether natural or anthropogenic, can affect parasite dynamics through separate pathways involving changes in host susceptibility, behaviour and/or demography. Furthermore, it is quite possible that several of these pathways act simultaneously within a given host–parasite system. Together, these changes can lead to either net increases or decreases in infection risk, and impact the epidemiological outcomes in opposite directions, depending on the balance of how each pathway is impacted by provisioning (Becker et al., 2015; Becker & Hall, 2014).

Given the many ways in which provisioning can affect infectious disease epidemiology, mechanistic models can be invaluable tools to explore how provisioning‐altered parameters relating to host demography, contact behaviour and host immune defence interact to affect the dynamics of parasite infection and spread. In particular, previous theoretical studies demonstrated that a range of parasite responses (i.e. changes in prevalence and basic reproductive number, *R*_0_) could arise from coupling functional responses of host birth and death, host susceptibility and contact rate parameters to the level of resource provisioning (Becker & Hall, 2014). For instance, increases in both host density and contact rates due to resource provisioning boost *R*_0_ (McCallum et al., 2001)_,_ when host immunity is not directly affected. However, if a host with access to improved nutritional resources was better able to protect itself from infection due a strengthened immune response, then the parasite's *R*_0_ would be significantly reduced. Additionally, parasite extinction could even occur at intermediate provisioning levels, with persistence only occurring at low or high provisioning levels (Becker et al., 2015), showing that parasite persistence changes along a gradient of resource provisioning, depending on the underlying effects of resources on the host. Therefore, modelling approaches are valuable tools for understanding the opposing patterns in disease outcomes observed in nature.

To date however, theory has focussed on a narrow subset of parasite types: typically directly transmitted microparasites that fit the classic ‘Susceptible‐Infected’ (SI) or ‘Susceptible‐Infected‐Recovered’ (SIR) frameworks. Given the diverse ways that provisioning can affect host physiology/immunology, contact patterns and/or host demography, we may expect the ultimate epidemiological consequences of provisioning to depend on key aspects of parasite life history, such as transmission mode or duration of infection. For example, if provisioning induces behavioural changes causing aggregations around high‐resource patches, then this may favour the transmission of contact‐transmitted parasites, but may have little impact on environmentally transmitted parasites, particularly if those resource patches are relatively transient in nature. We may also expect demographic responses to provisioning that increase host population size to have major consequences for parasites that show density‐dependent transmission, but little effect on parasites that exhibit frequency‐dependent transmission. Alternatively, provisioning effects that increase host survival may have little impact on acutely infecting parasites which are cleared before hosts typically die, but may have significant consequences for the fitness of chronic parasites. Furthermore, how macroparasites, rather than microparasites, are impacted by provisioning of the host remains poorly understood, as impacts on burden‐dependent processes (Anderson & May, 1978; Dobson & Hudson, 1992) may introduce epidemiological outcomes not seen for microparasites. To date, we have little understanding of how such diverse parasite types, beyond those conforming to standard SI or SIR frameworks, are likely to respond to host resource provisioning.

A well‐studied host system, which harbours a diverse array of microparasites and macroparasites with differing transmission modes and life histories, is the wood mouse *Apodemus sylvaticus*. The parasite communities of this species comprise various helminth, viral, protozoal and bacterial parasites, all of which have been intensively studied for a number of decades (Carslake et al., 2006; Erazo et al., 2021; Knowles et al., 2012; Knowles et al., 2013; Sweeny, Albery et al., 2021; Telfer et al., 2002; Telfer et al., 2007; Withenshaw et al., 2016). Here we use data from UK wood mouse parasite communities to develop and parameterise a series of epidemiological models to explore how these different parasite types may vary in their overall responses to host resource provisioning, within the same host population. Specifically, we developed and parameterised five models representing parasites from the wood mouse system that vary in their biology (microparasite and macroparasite), transmission mode (direct, environmental and vector transmitted) and duration of infection (acute, latent and chronic), and explored how the predicted responses to the same provisioning scenarios varied across this suite of parasites. These analyses show both the generalities of response to provisioning that hold across a range of parasite types, but also highlight the importance of identifying specific aspects of host and parasite biology, to properly infer the effects of provisioning for any specific host–parasite system.

## 2 MATERIALS AND METHODS

### 2.1 Model construction

**FIGURE 1:**
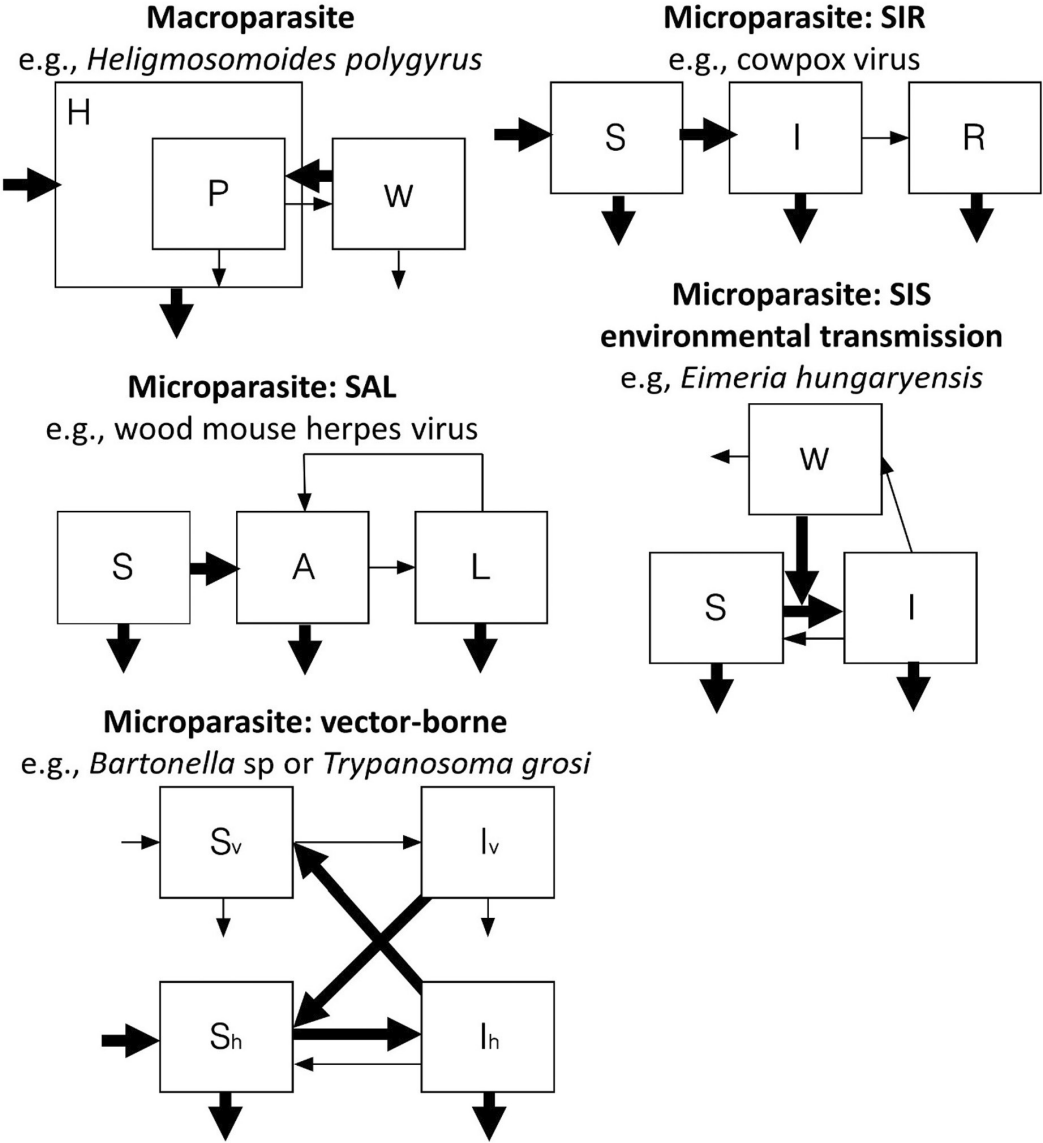
Schematic diagrams of the epidemiological modelling frameworks, each corresponding to the different exemplar species in the wood mouse parasite community (Table 1). Boxes represent population classes in each system. For microparasite models, hosts were classified by their infection status, where S, I, R, A and L represented susceptible, infected, recovered, acute and latent classes, respectively. For the SAL model, we explicitly include male and female classes, motivated by our previous work on wood mouse herpes virus in this system (Erazo et al., 2021). For the vector‐borne parasite, the subscripts *v* and *h* denote classes relating to the vector and host populations, respectively; note we assume density‐dependent, rather than frequency‐dependent vector transmission, as this was strongly supported by our model fitting (see Supplementary Material). For the macroparasite model, only one host class (H) was considered, *P* represents the size of the adult parasite population and *W* represents the size of the environmental pool of infective stages. Similarly, *W* represents the environmental pool of infective stages for the SIS model. Thick arrows denote processes that we allow to be affected by resource availability (affecting either contact rates, host susceptibility or host demography).

Based on common parasites from the UK wood mouse system (Table 1), we constructed five compartmental models, each describing a different, but common parasite type: (a) macroparasite, (b) microparasite SIR (susceptible–infected–recovered), (c) environmentally transmitted microparasite SIS (susceptible–infected), (d) sex‐biased microparasite SAL (susceptible–active–latent, with different potential transmission rates within and between male and female classes; Erazo et al., 2021); and (v) vector‐borne microparasite SIS (Figure 1). While we seek to model a diverse range of parasite types, the list we consider cannot be exhaustive. In particular, we do not consider trophically transmitted parasites, partly because we do not have data on such parasites from our exemplar wood mouse system, and partly because to do so would require consideration of how resource provisioning may independently affect the demography, behaviour and physiology of both host species, thereby greatly increasing the complexity of the models. However, a dedicated analysis of this mode of transmission would be a valuable addition to our broader understanding of provisioning effects on parasites more generally.

**TABLE 1:** Parasites from the *Apodemus sylvaticus* system: Model, type, transmission mode, diagnostic, mean faecal egg count or prevalence across the full data set and parameter values. *K =* 42.12* in all parasite models. α is the host‐to‐parasite contact rate, 𝛿 is the host susceptibility (probability of successful infection given contact). See Supplementary Material for details of parameter estimation. *Baseline parameter values. Note, we arbitrarily partition the estimated transmission rates into the baseline $\alpha_{\min}$ and $\delta_{\max}$ values shown, but emphasise our results are not sensitive to the specific values used (see Supplementary Materials for details).

Parasite (model)	Type	Transmission mode	Diagnostic	Mean FEC or prevalence	$\alpha_{\min}$ ρ=0*	$\alpha_{\max}$ ρ=1	$\delta_{\min}$ ρ=1	$\delta_{\max}$ ρ=0*
*Heligmosomoides polygyrus* (macroparasite)	Nematode	Environmental	Faecal egg count (FEC)	37.2 EPG	0.001	0.002	0.103	0.205
Cowpox virus (SIR)	Virus	Contact	Seroprevalence	3.7%	0.1	0.2	0.045	0.090
Herpesvirus (SAL)	Virus	Contact	Seroprevalence	14.7%	0.1	0.2	Male to male: 0.128 Female to male: 0.212 Male to female: 0.074 Female to female: 0.078	Male to male: 0.255 Female to male: 0.424 Male to female: 0.148 Female to female: 0.156
*Eimeria hungaryensis* (SIS)	Coccidian	Environmental	Faecal egg count	25.7%	0.00001	0.0002	0.048	0.096
*Bartonella* spp. (vector‐borne)	Bacteria	Vector	PCR	52.1%	0.01	0.02	0.408	0.815
*Trypanosoma grosi* (vector‐borne)	Protozoan	Vector	PCR	10.7%	0.01	0.02	0.053	0.106

The specific details of each model are given in the Supplementary Material, but they all followed standard structures well known from the epidemiological literature (e.g. Anderson & May, 1978, 1981, 1991). Here we illustrate our model construction using the familiar SIR framework (see also Supplementary Material):

(1) $$\frac{dS}{dt}=b\;N\left(1-\frac{N}{K}\right)-\alpha \delta \mathrm{SI}-\mu S,$$

(2) $$\frac{dI}{dt}=\alpha \delta \mathrm{SI}-\mathrm{\gamma I}-\mu I,$$

(3) $$\frac{\mathrm{dN}}{\mathrm{dt}}=bN\left(1-\frac{N}{K}\right)-\mu N,$$

where *S* and *I* are the densities of susceptible and infected hosts, respectively, and *N* is the total host population size (note the density of recovered individuals can be obtained from *R* = *N‐S‐I*), *b* and *μ* are host birth and death rates, respectively, and for simplicity we refer to *K* throughout as the host's carrying capacity, although we note that strictly it is the population size at which fecundity becomes zero, rather than the equilibrium disease‐free population size: $K\left(1-\mu /b\right)$. *γ* is host recovery rate. We break the standard transmission parameter, *β*, into two separate processes determined by the parameters α, the per capita contact rate, and δ, host susceptibility (the probability of infection given contact). The parameters in bold in Equations 1–3 (and the equivalents for the other model structures; see Figure 1 and Supplementary Material) represent the three potential routes by which resource provisioning can affect the epidemiology of the parasite: (1) host demography (through long‐term changes to the host's carrying capacity, *K*, or host birth (*b*) or death rates (*μ*)), (2) contact behaviour (α), and (3) immune defence (through changes to host susceptibility, δ). In what follows we assumed the host's carrying capacity (K), birth rate (*b*) and contact rate (α) would increase with resource provision to the host, while host susceptibility (δ) and mortality rate (*μ*) would decrease as hosts had more access to additional resources [as in (Becker & Hall, 2014) and (Becker et al., 2015)].

### 2.2 Incorporating resource provisioning

**FIGURE 2:**
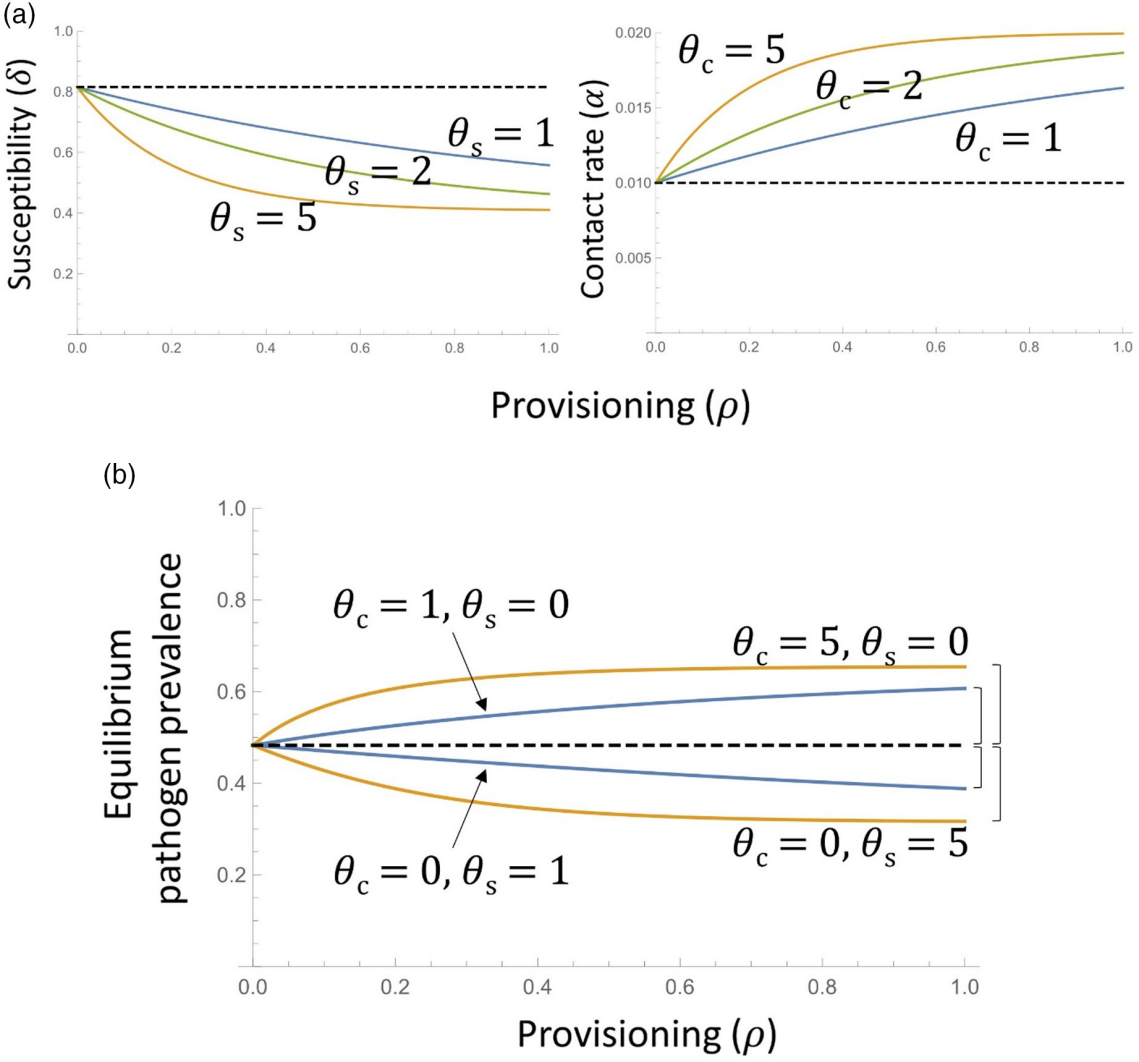
Illustration of how provisioning effects were incorporated into the models, and the quantification of their epidemiological effect. (a) Decreasing (for host susceptibility, 𝛿) and increasing (for contact rate, α) effects of increased provisioning (𝜌; *x*‐axis). The different coloured lines show the responses due to different levels of the ‘sensitivity’ parameters ($\theta_{s}$ and $\theta_{c}$, respectively), and the dashed horizontal line shows the baseline effect in the absence of provisioning. (b) Relationship between provisioning rate and equilibrium pathogen prevalence (here using parameter values from the *Bartonella* model for illustrative purposes), for different combinations of the sensitivity parameters ($\theta_{c}$ and $\theta_{s}$); again, the dashed horizontal line represents the baseline prevalence in the absence of provisioning. The brackets on the right‐hand side illustrate the magnitude of the difference between the equilibrium prevalence at maximal provisioning (when 𝜌 = 1) and the baseline prevalence (when 𝜌 = 0), for each combination of sensitivity parameters; it is these difference values that we use to quantify the epidemiological consequences of the different provisioning scenarios explored.

To capture the effects of resource provisioning on both host contact rate and susceptibility, we followed the approach developed by Becker and Hall (2014), where provisioning is represented by the parameter ρ. We emphasise that we use the term ‘provisioning’ to refer to any increase in resource availability, whether naturally occurring or anthropogenically derived. Here ρ=0 represents no resource provisioning (hence, all parameters are at baseline values), while increasing values of ρ correspond to increasing levels of provisioning, for example due to human‐provided food sources, land‐use change increasing access to novel resources or periodic access to resources, such as through tree masting. As in Becker and Hall (2014)), parameter responses to provisioning were assumed to have a monotonic and saturating behaviour that depends on the parameter's sensitivity to provisioning, represented by $\theta_{x}$, allowing parameters to scale from no effect of provisioning when $\theta_{x}$ = 0, through to a quickly saturating relationship at high values of $\theta_{x}$ (see Figure 2a for illustrative examples of these relationships). Since the contact rate between hosts (α) is expected to increase with provisioning (Becker et al., 2015; Boutin, 1990), we used the following functional form:

(4) $$\alpha =\alpha_{\max}-\left(\alpha_{\max}-\alpha_{\min}\right)e^{-\theta_{c}\rho }.$$

Because susceptibility to infection (δ) is hypothesised to decrease with provisioning ((Becker et al., 2015; Sweeny, Clerc et al., 2019), we used the following function:

(5) $$\delta =\delta_{\min}+\left(\delta_{\max}-\delta_{\min}\right)e^{-\theta_{s}\rho },$$

where $\alpha_{\min}$ and $\delta_{\min}$ are the minimum values, and $\alpha_{\max}$ and $\delta_{\max}$ are the maximum values that contact rate and susceptibility could take (Figure 2a). Thus, if ρ=0, $\delta =\delta_{\max}$ and $\alpha =\alpha_{\min}$, the parameter baseline values. In an intensive resource provisioned scenario (high ρ), the maximum contact rate was assumed to be twice the baseline value ($\alpha_{\max}=2\alpha_{\min}$) and the minimum susceptibility was assumed to be half the baseline value ($\delta_{\min}=\frac{\delta_{\max}}{2}$).

### 2.3 Model parameterisation

We parameterised each model using a specific parasite species from our 4‐year longitudinal dataset of 1,453 individually tagged wood mice *Apodemus sylvaticus* captured across four 70 × 70 m grids, at Haddon Wood on the Wirral, UK between June 2009 and December 2012 (Knowles et al., 2012; Sweeny, Albery et al., 2021). Wood mice in these populations are commonly infected by five different species of parasites which corresponded to the different modelling frameworks described above (see Table 1 for details): *Heligmosomoides polygyrus* (macroparasite), cowpox virus (SIR), Wood Mouse Herpes Virus (WMHV; SAL)*, Eimeria hungaryensis* (SIS)*, Bartonella* spp. (vector‐borne), *Trypanosoma grosi* (vector‐borne).

Full details of model parametrisation are provided in the Supplementary Material but, briefly, parameters were estimated using adaptive Monte Carlo Markov Chain Metropolis‐Hastings (MCMC‐MH) using the R package fitR (Camacho & Funk, 2019), and assuming uniform priors. Model fitting was carried out in two stages. First, host demographic parameters were estimated by fitting the predicted total number of mice per week to the observed number of mice captured per week. Next, we estimated parasite infection‐related parameters for the various parasite models (see Table 1), through fitting each model individually to relevant data on infection status for that parasite species. For the microparasite models, we used data on seroprevalence for Wood Mouse Herpes Virus (Knowles et al., 2012) and PCR diagnostics for *Trypanosoma grosi* and *Bartonella* spp. (Knowles et al., 2013; Withenshaw et al., 2016). We used faecal egg or oocyst counts (measured as eggs or oocysts per gram of faeces) for the gastrointestinal nematode *H. polygyrus* and the coccidian *E. hungaryensis*, respectively (Knowles et al., 2013). Because the seroprevalence of cowpox was very low in our dataset (<5%), the infection‐related parameters were estimated using data from previous literature (Telfer et al., 2002); see Supplementary Material.

### 2.4 Quantifying effects of resource provisioning

The estimated parameter values (Table 1) were assumed to represent the baseline values ($\delta =\delta_{\max}$ and $\alpha =\alpha_{\min}$) corresponding to the scenario of no resource provisioning (ρ=0). Under the scenario of arbitrarily intensive resource provisioning (ρ=1), parameter values for δandα were calculated using Equations 4 and 5 (Becker & Hall, 2014). With this framework, we then investigated the net effect of host resource provisioning for each of the above parasite types as the absolute difference in either mean parasite burdens for macroparasites or parasite prevalence for microparasites at equilibrium, between these two provisioning scenarios (intensive provisioning, ρ=1, and no provisioning, ρ=0; see Figure 2b for illustration of this process). Here a difference in those equilibrium metrics greater than 0 implies a net increase in overall prevalence or mean parasite burden due to provisioning, whereas a value <0 implies a net reduction due to provisioning. All equilibrium values were calculated numerically using Mathematica v. 12.1.

We then explored how these changes for each parasite depended on the balance of the host susceptibility and host contact sensitivities to resource provisioning. We did this by exploring within $\theta_{s}$ – $\theta_{c}$ parameter space, with each of those sensitivity terms varying from 0 (no response of the relevant parameter to provisioning), up to an arbitrarily high value of 5 (rapid progression to the maximum or minimum value of the relevant parameter under provisioning). To simplify the analysis and presentation, we incorporated the effects of provisioning on host demography by varying host carrying capacity from K to 2K, to reflect long‐term increases in overall food availability due to increased provisioning (Becker et al., 2015; Ozoga & Verme, 1982). We also explored alternative demographic responses through long‐term changes in either host birth rates (*b*, assumed to increase with provisioning) or host mortality rates (μ, assumed to decrease with provisioning). Overall, these analyses allowed us to explore how the overall changes due to provisioning vary with the sensitivity of the individual host demographic, contact rate or susceptibility parameters to provisioning.

The present study did not require ethical approval.

## 3 RESULTS

**FIGURE 3:**
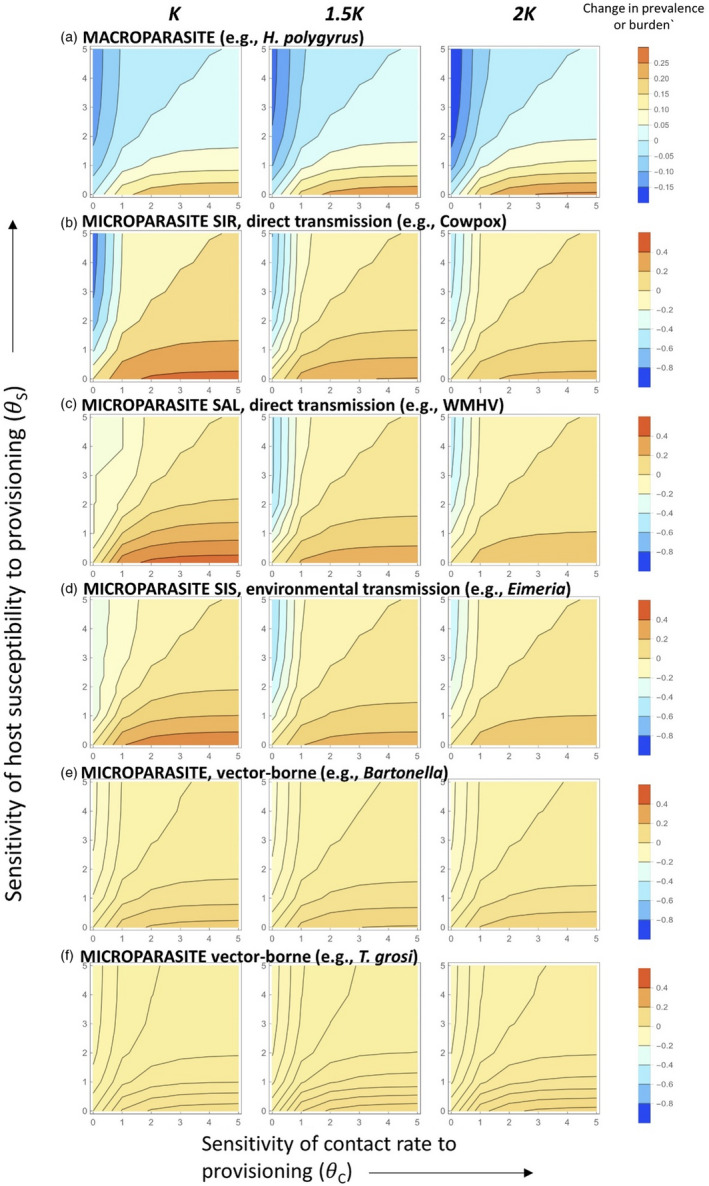
Parasite outcome changes induced by provisioning. Row a shows changes in macroparasite mean burden and rows b–f represent changes in microparasite prevalence (the differences in equilibrium prevalence between provisioned (ρ=1) and the un‐provisioned baseline (ρ=0) scenarios). The colours show the magnitude of those differences; increasing redness shows provisioning has an increasingly positive effect on that parasite's burden or prevalence, increasing blueness shows provisioning has an increasingly negative effect on burden or prevalence. To facilitate comparison of the absolute magnitudes of the effects, the colour‐coding scale is consistent across all panels and microparasite species (except for row A, the macroparasite, which shows changes in mean worm burdens rather than prevalence). For all figures, the *X‐*axis represents the sensitivity of contact rate to provisioning $\left(\theta_{c}\right)$ and *y‐*axis is the sensitivity of host susceptibility to provisioning $\left(\theta_{s}\right)$. Columns illustrate different carrying capacities, varying from K to 2K, representing increasing levels of host demographic response to provisioning.

For all parasite species examined, we found the same qualitative responses of parasite infection (either prevalence or mean burden) to host resource provisioning; all parasite infection measures increased when contact rate was highly sensitive to provisioning ($\theta_{c}\to 5$, the maximum level explored) and host susceptibility did not change under provisioning ($\theta_{s}=0$; red regions, Figure 3; Figure S1). Hence, as may be expected, rapid increases in host contact rate due to provisioning (high $\theta_{c}$), coupled with a negligible response of host immunity to provisioning (low $\theta_{s}$), result in high levels of infection for all parasite types. Conversely, prevalence and infection burden for all parasites was most reduced in the opposite scenario, when host susceptibility was highly sensitive to provisioning and contact rate was not affected by provisioning ($\theta_{c}=0$, $\theta_{s}\to 5$; blue regions, Figure 3; Figure S1). Hence, as expected, rapid increases in immunity due to provisioning (high $\theta_{s}$), coupled with negligible changes to contact rate (low $\theta_{c}$), result in low levels of infection under provisioning, and this was true across all parasite types.

However, although all parasites showed the same qualitative responses to provisioning, the different parasite types differed considerably in their quantitative responses. For clarity of presentation, we initially describe these results assuming no host demographic change to provisioning (carrying capacity fixed at the baseline value of *K*; Figure 3 left‐hand column), but below we consider the effects of incorporating longer term host demographic changes due to resource provisioning, either through changes in the carrying capacity, or host birth or death rates. In the absence of host demographic responses to provisioning, the directly transmitted SIR microparasite (cowpox; Figure 3, row B) was predicted to show the strongest response overall to provisioning, with ~40% predicted increase in prevalence due to provisioning for high $\theta_{c}$ (rapid increase in contact rate due to provisioning) and low $\theta_{s}$ (negligible change in immunity due to provisioning), and ~80% predicted decrease in prevalence due to provisioning for low $\theta_{c}$ and high $\theta_{s}$. The SAL and SIS microparasites (WMHV and *E. hungaryensis*, respectively) were also predicted to show reasonably strong (~20%–30%) increases in prevalence due to provisioning for high $\theta_{c}$ and low $\theta_{s}$, but little change in prevalence for all other combinations of parameters (Figure 3, rows C, D). Comparatively, the two vector‐borne parasites (*Bartonella* and *T. grosi*) were predicted to show very minor variations in prevalence (±10%) due to provisioning across all combinations of $\theta_{c}$ and $\theta_{s}$ (Figure 3, rows E, F). We cannot directly compare the magnitude of responses by the macroparasites with the other parasites, since they are measured in different ways (changes in mean worm burdens due to provisioning vs. changes in prevalence). However, our model predicts maximum absolute changes in predicted mean abundance of the macroparasite were very low (~0.25; Figure 3, row A), suggesting predicted mean worm burdens are very robust, showing limited effects of provisioning overall.

When we included the effect of longer term host demographic responses to provisioning, first modelled as fixed changes in host carrying capacity, the varying parasite types responded differently. As carrying capacity was increased from *K* to 2 *K*, the magnitude of predicted changes in macroparasite (*H. polygyrus*) mean burden due to provisioning intensified (Figure 3, row A); with both increases in burden under high contact rate sensitivity ($\theta_{c}\to 5$), and decreases in burden under high susceptibility sensitivity ($\theta_{s}\to 5$), predicted to become intensified with higher host carrying capacities (although as noted, the absolute changes in mean burden remained very small). Conversely, the opposite pattern was predicted to occur for the microparasite SIR model (cowpox), where the change in prevalence became less dramatic as carrying capacity increased (e.g. whereas high $\theta_{c}$ was associated with >30% predicted increase in prevalence when the carrying capacity was kept at *K* (Figure 3; Figure S1 row B, left‐hand plot), increasing the carrying capacity to 2 *K* resulted in ~20% predicted increase in prevalence (Figure 3; Figure S1 row B, right‐hand plot). The SAL and SIS models (WMHV and *E. hungaryensis*, respectively) were predicted to show mixed responses to rises in carrying capacity depending on the different sensitivity parameters ($\theta_{c}$ and $\theta_{s}$); the magnitude of positive changes in prevalence due to provisioning (i.e. in the region of high $\theta_{c}$ and low $\theta_{s}$) was neutralised as carrying capacity increased for both parasites (Figure 3; Figure S1 rows C and D), whereas the region of negative changes in prevalence due to provisioning (i.e. in the region of low $\theta_{c}$ and high $\theta_{s}$) initially intensified as carrying capacity was increased from *K* to 1.5 *K*, but then diminished as it was increased further to 2 *K*. The two vector‐borne parasites however were largely unresponsive in absolute terms to changes in host carrying capacity due to provisioning (Figure 3; Figure S1, rows E and F).

Finally, we considered alternative demographic impacts of provisioning, either increasing host birth rate (*b*; Figure S2) or decreasing host mortality rate (*μ*; Figure S3). Broadly, none of the parasites were predicted to show notable changes in their responses to provisioning due to changes in host birth rates (Figure S2). Furthermore, neither the macroparasite SIR model nor, as with demographic changes acting on the host's carrying capacity, the vector‐borne pathogens were predicted to show notable response to provisioning effects on host death rates (Figure S3, rows A, B, E and F). However, the SAL and SIS models predicted an intensification of the negative response to provisioning for high $\theta_{s}$ values with reducing host mortality rates (Figure S3, rows C, D).

## 4 DISCUSSION

Existing theory on the responses of parasites to resource provisioning of their hosts has tended to focus on a relatively limited range of parasite types: microparasites conforming to SIR or SI‐type dynamics (Becker & Hall, 2014). However, although our analyses predicted common general responses for a range of parasites infecting UK wood mice, matching that previous theory (Altizer et al., 2018), we also predicted notable differences, particularly in the magnitude of responses by the different parasites. Hence, specific epidemiological outcomes could strongly depend on parasite type (microparasite or macroparasite), the duration and long‐term outcome of infection (whether SIS, SIR or SAL), and whether the parasite is directly, environmentally or vector‐borne transmitted. Although we focus specifically on the UK wood mouse parasite community as our exemplar system for these analyses, to ensure our predictions have a biological grounding, these findings have general relevance in illustrating that different parasites may exhibit different responses to the same degree of resource provisioning in the same host population. As such, these analyses highlight the importance of understanding key aspects of host–parasite biology to properly infer the effects of resource provisioning, whether through natural events or human activities, on host–parasite interactions.

Prevalence was predicted to increase substantially under provisioning for each of the directly transmitted and environmentally transmitted microparasites examined (Figure 3, rows B, C, D), when the response of host contact behaviour to provisioning ($\theta_{c}$) is high and the response of host immunity ($\theta_{s}$) is low. An example of this may be seen in European greenfinches *Chloris chloris*, where aggregation around bird feeders increases transmission of *Trichonomas gallinae* (Lawson et al., 2012). Similarly in Elk *Cervus elaphus*, supplemental feed stations increased contact rates, leading to higher *Brucella abortis* exposure (Cross et al., 2007). In contrast, prevalence was predicted to decline due to provisioning if host susceptibility is affected by improved nutrition more than contact rate is. An example of this may be seen in lace monitors *Varanus varius* foraging on human garbage, where it was shown that improved body condition was associated with lower intensity of blood parasites compared to non‐provisioned individuals (Jessop et al., 2012). Another example of this scenario comes from field experiments on our exemplar system, which showed that resource‐provisioned wood mice were less susceptible to *H. polygyrus* infection, cleared worms more efficiently and maintained better body condition than un‐provisioned mice (Sweeny,Clerc et al., 2019). These examples suggest that increased host aggregation, and therefore increased transmission, is not always an outcome of provisioning. Indeed, changes in host contact rates due to resource provisioning are likely to depend on the spatial arrangement of resources; when food is aggregated, contact rate between hosts could increase. This may be happening, for example, for urban feral cats, which show smaller foraging ranges and higher territory overlap compared to rural cats (Schmidt et al., 2007). However, if food is more evenly distributed, contact rate could actually decrease with provisioning. Spatially explicit models could be useful for studying how resource provisioning modifies contact rates and therefore parasite persistence (Plowright et al., 2011).

When we explored long‐term host demographic effects of provisioning, either acting on the host's carrying capacity, birth rate or death rate, we generally found either an overall weakening of responses compared to when those demographic parameters were held at their baseline level, or very little change. An exception to this was the SAL model, where reducing host death rate with increased provisioning tended to exacerbate reductions in prevalence due to the reduction in susceptibility (Figure S3, row C, high $\theta_{s}$ values). Similarly for the macroparasite model, increasing the host's carrying capacity also exacerbated reductions in work burdens due to reduced susceptibility (Figure 3, row A), although in general the macroparasite model predicted very limited absolute changes in mean worm burdens under any of the provisioning scenarios examined, suggesting high stability for this system. Limited epidemiological response to demographic changes under resource provisioning is supported by observations from larger vampire bat *Desmodus rotundus* colonies in livestock areas, where provisioning was found to increase host population size, but no effect of rabies virus exposure was observed (Streicker et al., 2012). However, our analyses did show that the environmentally transmitted parasite that does not confer lasting immunity (e.g. *E. hungaryensis*, represented by the SIS model) was most suppressed due to provisioning at the intermediate carrying capacity examined (i.e. Figures 3; Figure S1, row D), particularly if host susceptibility rapidly declines with provisioning (high $\theta_{s}$). This finding supports previous research suggesting that at an intermediate demographic response to provisioning, parasite persistence (as measured by its *R*_0_) attains its minimum, although susceptibility would have to be more affected by provisioning compared contact rate (Becker et al., 2015).

In this study, we assumed that any impact of resource provisioning on host physiology would be positive, and therefore decrease host susceptibility to parasite infection. However, this may not always be the case. For example, it is important to consider both resource quantity and quality; although resource quantity might increase, for example through increased availability of human garbage, quality could be poorer compared to a baseline scenario, thereby increasing host susceptibility to infection (Maggini et al., 2007; Van Heugten et al., 1996). For instance, rock iguanas feeding from carbohydrate‐rich food sources provided by tourists had higher hookworm burdens compared to non‐supplemented individuals (Knapp et al., 2013). Furthermore, we do not explicitly consider the possibility that increased resources may directly benefit the parasites by increasing within‐host replication rate and hence infectiousness (Cressler et al., 2014). Phenomenologically, however, such an effect may be envisaged as a reduced responsiveness of host susceptibility due to provisioning (i.e. any boosting of the host's immune response due to increased resources may be counteracted by a boosting of pathogen growth, resulting in a net reduction of $\theta_{s}$). Such reduction in $\theta_{s}$ could also be thought as an outcome of the host adopting a ‘tolerance’, rather than resistance, response to infection (Råberg et al., 2009; Vale et al., 2014). Exploring the outcomes of these scenario more fully though would require explicit consideration of the balance of these separate within‐host processes, which is beyond the scope of the current paper. Finally, we note that, although we considered a range of parasite types, we did not consider all possible life cycles and, in particular we ignored trophically transmitted parasites with complex life cycles involving intermediate hosts. Such systems would require consideration of how those alternative host species themselves respond to resource provisioning, resulting in more complex net responses of such parasites to provisioning. Future theoretical studies on the role of provisioning on parasite transmission more generally could therefore involve even more complex systems than those considered here.

In conclusion, we demonstrate that although there are intuitive, general responses to resource provisioning that are consistent across a wide range of parasite types, we also show that different parasite species infecting the same host species can show markedly different responses to the same provisioning scenarios. Understanding the key parasite characteristics and host pathways driving these patterns is important, as resource provision can impact infectious disease dynamics in opposite directions. Hence, it is crucial to recognise system‐specific variations, such as specific host behaviours at the individual level, parasite interactions with the host immune system, and how resource availability shapes host demographics, to fully understand and predict epidemiological responses to provisioning.

## AUTHORS' CONTRIBUTIONS

D.E. conducted the analyses and led the writing of the manuscript; D.E. and A.F. developed the modelling approaches; A.B.P. and A.F. generated the data as part of previous grant funding (see Acknowledgements for funding details). All authors contributed critically to the drafts and gave final approval for publication.

## CONFLICT OF INTEREST

The authors have no conflicts of interest to declare.

## Supporting information


Appendix S1

## Data Availability

Data available from Zenodo Digital Repository https://doi.org/10.5281/zenodo.6573377 (Erazo et al., 2022a) and https://github.com/dieraz/prov‐theo.git (Erazo et al., 2022b).
