# Supplementary material for: The predicted impact of resource provisioning on the epidemiological responses of different parasites
Source: J Anim Ecol. 2022 Jun 14;91(8):1719–30. doi: 10.1111/1365-2656.13751 (PMC9546467; doi:10.1111/1365-2656.13751)
Supplement: Supplementary file 1 — Appendix S1 [file JANE-91-1719-s001.docx]

**The predicted impact of resource provisioning on the epidemiological responses of different parasites**

**Diana Erazo, Amy B. Pedersen, Andy Fenton**

**Supplementary material**

*Model frameworks*

Each model had, at its heart, the same host demographic model:

$\frac{dN}{dt}=bN\left( 1-\frac{N}{K} \right)-\mu N$ Eq S1

where$N$ represents the total host population, $K$ the carrying capacity, $b$the birth rate and, $\mu$ the mortality rate; in what follows we assumed infections do not increase host mortality. For our analyses, we used this demographic model as a template, and developed five compartmental models, each reflecting different parasites types (inspired by our field system; described below), to study the effects of host resource provisioning on the dynamics of each parasite. Each model incorporated parameters reflecting contact rate ($\alpha$) and host susceptibility ($\delta$), that were allowed to vary with provisioning (see main text).

The **macroparasite model** keeps track explicitly of the number of macroparasites $(P$) within hosts $(H$), which reproduce and release larvae $(W$) into the environment. Adult parasites $(P$) are assumed to be acquired when hosts $(H$) contact the parasite free-living stage in the environment at rate $\alpha$, and which establish with probability $\delta$; hence the net rate of acquisition of new infections occurs at rate $\alpha\delta WH$. $P$ decreases by two factors: host natural mortality ($\mu$) and host parasite-induced mortality ($\nu P$) dependent on the mean burden of worm infection. For macroparasites, parasite aggregation or crowding is incorporated via the term $\frac{P^{2}\nu(k+1)}{Hk}$ where $k$ represents the aggregation coefficient (exponent of the negative binomial distribution) (Anderson & May, 1978). The environmental pool of larvae $(W$) increases when the adult parasites $(P$) release larvae at $\tau$ rate and decreases due to death at rate $\phi$. The macroparasite model is therefore represented by a three-equation system as follows:

$\frac{dH}{dt}= b H\left( 1-\frac{N}{K} \right)-\mu H-\nu P$ Eq S2

$\frac{dP}{dt}= \alpha\delta WH-\left( \mu+\nu\right)P-\frac{P^{2}\nu\left( k+1 \right)}{Hk}$ Eq S3

$\frac{dW}{dt}= \tau P-\phi W$ Eq S4

Our first microparasite model is the **SIR model**, in which $S$, $I$, and $R$ represent the susceptible, infected and recovered host populations. Susceptible hosts become infected when contacting infected hosts, according to the term $\alpha\delta SI$, where $\alpha$ is the per capita counter rate between susceptible and infected hosts, and $\delta$ is host susceptibility or the probability that the contact results in infection. Infected individuals recover to life-long immune hosts at rate $\gamma$. The SIR model is therefore represented by a two-equation system (where the dynamics of the *R* class are implicit, as *N* = *S+I+R*), as follows:

$\frac{dS}{dt}= b N\left( 1-\frac{N}{K} \right)-\alpha\delta SI-\mu S$ Eq S5

$\frac{dI}{dt}= \alpha\delta SI-\gamma I-\mu I$ Eq S6

For the **sex-biased microparasite SAL model**, as motivated by a general herpesvirus system (Erazo, Pedersen, Gallagher, & Fenton, 2021), susceptible individuals ($S$) become infected and enter the active class ($A$) through contact with active-infected individuals (latent-infected individuals are assumed not to transmit infections) at transmission rate $\alpha\delta$, where $\alpha$ is the per capita contact rate between susceptible and acute hosts, and $\delta$ is host susceptibility or the probability that the contact results in infection. Active-infected individuals then move to the latent class ($L$) at transition rate $\varepsilon$ (hence active infections last on average 1/$\varepsilon$ weeks), and vice versa at rate $\eta$ (hence latent infections last on average 1/$\eta$ weeks). We assumed individuals do not recover from infections.

The model considered explicit female and male classes and density-dependent transmission for all possible transmission routes ($\beta_{1}-\beta_{4}$). Therefore, the model consisted of six classes (susceptible, active-infected and latent-infected, for both females and males), and all transmission terms had the form ${\alpha_{n}\delta}_{n}S_{i}A_{j}$ where $i$ and $j$ represent sex, either female or male, and ${\alpha_{n}\delta}_{n}$ is the transmission term between $i$ and $j$. The sex-biased microparasite model is therefore represented by a six-equation system as follows:

$$\frac{dS_{m}}{dt}= b \frac{N}{2}\left( 1-\frac{N}{K} \right)-\alpha_{1}\delta_{1}S_{m}A_{m}{-\alpha}_{2}\delta_{2}S_{m}A_{f}-\mu S_{m}$$

 Eq S7

$\frac{dA_{m}}{dt}=\alpha_{1}\delta_{1}S_{m}A_{m}{+\alpha}_{2}\delta_{2}S_{m}A_{f}-\mu A_{m}-\varepsilon A_{m}+\eta L_{m}$ Eq S8

$\frac{dL_{m}}{dt}=\varepsilon A_{m}-\eta L_{m}-\mu L_{m}$ Eq S9

$\frac{dS_{f}}{dt}= b \frac{N}{2}\left( 1-\frac{N}{K} \right)-\alpha_{3}\delta_{3}S_{f}A_{m}{-\alpha}_{4}\delta_{4}S_{f}A_{f}-\mu S_{f}$ Eq S10

$\frac{dA_{f}}{dt}=\alpha_{3}\delta_{3}S_{f}A_{m}{+\alpha}_{4}\delta_{4}S_{f}A_{f}-\mu A_{f}-\varepsilon A_{f}+\eta L_{f}$ Eq S11

$\frac{dL_{f}}{dt}=\varepsilon A_{f}-\eta L_{f}-\mu L_{f}$ Eq S12

For the **microparasite environmentally-transmitted SIS model**, as motivated by an *Eimeria* (gastrointestinal coccidia) parasite (Table 1, main text), hosts get infected when ingesting eggs in the environment ($W$), according to the term $\alpha\delta SW$, where $\alpha$ is the per capita contact rate between hosts and eggs, and $\delta$ is host susceptibility or the probability that the contact results in infection. Each infected individual sheds eggs at rate $\tau$ and recovers (back to being a fully susceptible host) at rate $\gamma$. Eggs in the environment could become inactive and lose the ability to infect susceptible hosts, thus $\phi$represents egg decay rate in the environment. The microparasite environmentally-transmitted (SIS) model is therefore represented by a three-equation system as follows:

$\frac{dS}{dt}= b N\left( 1-\frac{N}{K} \right)-\alpha\delta SE+\gamma I-\mu S$ Eq S13

$\frac{dI}{dt}= \alpha\delta SW-\gamma I-\mu I$ Eq S14

$\frac{dE}{dt}= \tau I-\phi W$ Eq S15

The **vector-borne SIS system** considers vector ($S_{v}$, $I_{v})$ and host populations ($S_{h}$, $I_{h})$, which are divided by their infection status. The vector population increases at rate $b_{v}N_{v}\frac{(K_{v}-N_{v})}{K_{v}}$ where $b_{v}$, $N_{v}$ and $K_{v}$ represent vector birth rate, total vector population size and vector carrying capacity, respectively. Susceptible vectors ($S_{v})$ become infected when having a successful contact with infected hosts $(I_{h})$, expressed by the $\alpha\omega S_{v}I_{h}$ term where $\alpha$ is the per capita contact rate between hosts and vectors, and $\omega$ is vector susceptibility. Vectors die at a $\mu_{v}$ rate. Susceptible hosts ($S_{h}$) become infected through contact with infected vectors ($I_{v})$ at vector-to-host transmission rate $\alpha\delta$, where again $\alpha$ is the host-to-vector contact rate, and $\delta$ is host susceptibility or the probability that the contact results in infection. Infected hosts $(I_{h})$ recover at a $\gamma$ rate. Note, this formulation assumes density-dependent transmission, rather than frequency-dependent, as is sometimes assumed for vector-borne pathogens; we tried both formulations in our model fitting (below), and the density-dependent version greatly out-performed the frequency-dependent version (DIC = 772 for density-dependent versus 27183 for frequency-dependent). We also note that our formulation allows provisioning to influence both host-to-vector and vector-to-host transmission, since both depend on the same contact rate (𝛼). The SIS vector-borne model is therefore represented by a four-equation system as follows:

$\frac{dS_{v}}{dt}= b_{v} N_{v}\left( 1-\frac{N_{v}}{K_{v}} \right)-\alpha\omega S_{v}I_{h}-{\mu_{v}S}_{v}$ Eq S16

$\frac{dI_{v}}{dt}= \alpha\omega S_{v}I_{h}-{\mu_{v}I}_{v}$ Eq S17

$\frac{dS_{h}}{dt}= b N_{h}\left( 1-\frac{N_{h}}{K_{h}} \right)-\alpha\delta S_{h}I_{v}-\mu S_{h}+\gamma I_{h}$ Eq S18

$\frac{dI_{h}}{dt}= \alpha\delta S_{h}I_{v}-\mu I_{h}-\gamma I_{h}$ Eq S19

*Field data collection for model parameterisation*

Data from individuals was generated longitudinally from wood mice (*Apodemus sylvaticus*) collected between June 2009 and December 2012 in four grids located in Haddon Wood, Cheshire, UK as described in (Knowles, Fenton, & Pedersen, 2012; Sweeny, Albery, Venkatesan, Fenton, & Pedersen, 2021). Two Sherman traps baited with grain and bedding were placed every 10 m in each 70 x 70 m squared grid. Trapping was conducted for 3 consecutive nights every 3 weeks during four field seasons: June-December in 2009 and 2012, and May-December in 2010 and 2011. All trapped individuals were tagged for recognition in subsequent recaptures. From all wood mice, morphometric measures were recorded, including sex and age.

The main parasite species in this system have been well characterised (Knowles et al., 2012, 2013; Sweeny et al., 2021; Withenshaw, Devevey, Pedersen, & Fenton, 2016), and are summarised in Table 1 (main text). Methods of identification of each parasite are described in the above references, but briefly, the GI parasites *H. polygyrus* and *E. hungaryensis* were identified from eggs or oocysts in faecal samples collected from the traps using the salt flotation technique; infection levels were quantified as faecal egg or oocyst counts (Knowles et al., 2013). The presence of microparasites were determined from blood samples taken from the tail at first capture within each month. Wood mouse Herpes Virus (WMHV) and cowpox infection was detected using a serological assay that detects antibodies in mouse serum using IFA (Knowles et al., 2012). Note, although seropositivity only measures whether an animal has been infected, not whether it has an on-going infection, it is valid to use as for WMHV as infections are lifelong, and we have previously shown it to correlate well with more direct measures of infection status (e.g., PCR; (Knowles et al., 2012)). For cowpox, seropositivity is not a reliable measure of active infection status, as animals can remain seropositive after clearing the infection (Smith et al., 2009). Hence, we cannot equate seroprevalence with the prevalence of animals with on-going infections (*I/N*). For that reason we assume seroprevalence corresponds to the prevalence of animals that had ever been infected: (*I+R*)/*N*. Bacteria of the genus *Bartonella*, and *Trypanosoma grosi* were screened and identified using PCR-based diagnostics (Withenshaw et al., 2016). All parasitological data was aggregated weekly. Mouse population size per week was defined as the total number of mice collected in the four grids during that period.

*Model parameterization*

Demographic parameters ($\sigma_{d}=\{K, \mu, b\})$ and parasite-related parameters ($\sigma_{p}$, which varied depending on the model type being fitted; see below) were estimated using adaptive Monte Carlo Markov Chain Metropolis-Hastings (MCMC-MH) (Camacho & Funk, 2019), assuming uniform priors, through fitting to data on wood mouse population abundances and infection seroprevalences or prevalence for microparasites and faecal/oocyst eggs counts for macroparasites. We ignored the first year (52 weeks) of predicted transient dynamics of the simulation as burn-in time, and fitted the models over the subsequent 4 years of data.

Model fitting was carried out in two stages. First, demographic parameters and carrying capacity were estimated by fitting the simulated total number of weekly wood mice (*l_mice_*; $N$ from Eqn S1) to the observed number of mice captured per week (*y_week_*). The weekly number of wood mice captured was assumed to follow a Poisson distribution. The log-likelihood of the data for the mice population dynamics model was given by:

$$l_{mice}(\sigma_{d})=l_{mice}\left( data | \sigma_{d} \right)=\sum_{week} l_{mice-week}(y_{week}|\sigma_{d})$$

Through this we generated posterior distributions for the carrying capacity, birth and death rate.

Next, we sought to estimate the different parasite-related parameters in the various parasite models. Models were parameterized from data on wood mice infected with five parasites: *Eimeria hungaryensis,* Wood Mouse Herpes Virus*, Bartonella* spp., *Trypanosoma grosi* and *Heligmosomoides polygyrus.* Note that the transmission rate for all parasites is the product of contact rate ($\alpha)$ and susceptibility ($\delta)$, thus for model fitting each of these products were considered as one single parameter for each parasite (the overall baseline transmission rate, $\bar{\beta}= \alpha\delta$); see below for how we then partitioned these values into separate contact rate and susceptibility components. Disease-related parameters estimated for each system were: *E. hungaryensis*:$\sigma_{p}=\{\alpha\delta,\gamma\}$, Herpesvirus:$\sigma_{p}=\{\alpha_{1}\delta_{1},\alpha_{2}\delta_{2},\alpha_{3}\delta_{3},\alpha_{4}\delta_{4}, \varepsilon,\eta\}$, *Bartonella* spp. and *T. grosi:*$\sigma_{p}=\{\alpha\omega,\alpha\delta,\gamma\}$, $=\{\alpha\delta,\gamma\}$, and *H. polygyrus:*$\sigma_{p}=\{\alpha\delta,\nu\}$. For the microparasites, the weekly simulated number of infected mice was fitted to the observed number of mice infected by that parasite per week. The observed number of infected mice were assumed to follow a Poisson distribution. The log-likelihood of the data is given by:

$$l\left( data | \sigma_{p} \right)=\sum_{week} l_{prev-week}\left( y_{week} | \sigma_{p} \right)$$

For the macroparasite, the weekly simulated total *H. polygyrus* larvae released by mice was fitted to the total larval counts in faecal samples per week. The number of larvae was assumed to follow a negative binomial distribution. The log-likelihood of the data is given by:

$$l\left( data | \sigma_{p} \right)=\sum_{week} l_{larvae-week}(y_{week}|\sigma_{p})$$

Three MCMC-MH chains of 10,000 iterations per model were run using the default parameter standard deviation (parameter value divided by 10). Then, for each chain, using the first chain output (standard deviation and $\bar{\theta}$for the last 9,000 iterations) as input, we ran a second chain of 100,000 iterations. For the second chain, the first 5,000 iterations were discarded (burn-in), and we eliminated every 10 samples per sample to avoid auto-correlation (thinning). The Gelman-Rubin diagnostic was used to assess MCMC convergence by analysing the difference between chains. Through this we first generated posterior distributions for the carrying capacity, birth and mortality rate. Using this demographic parameter estimates, we then generated posterior distributions for disease-related parameters for each parasite system individually.

For our provisioning analyses we needed to partition the estimated composite transmission parameters (the $\bar{\beta}$) into the individual contact rate and susceptibility parameters ($\alpha$ and $\delta$, respectively), because resource provisioning may induce different responses in direction and magnitude for the processes separately. Importantly, our results are not sensitive to how we do this partitioning. To show this, replacing $\alpha$ and $\delta$ with our assumed provisioning-dependent functional forms (Eqs 4 and 5 from the main paper), the overall transmission rate in each model becomes:

$$\alpha\delta=\left[ \alpha_{max}-(\alpha_{max}-\alpha_{min})e^{-\theta_{c}\rho} \right]\left[ \delta_{min}+(\delta_{max}-\delta_{min})e^{-\theta_{s}\rho} \right]$$

which, with our assumptions that $\alpha_{max}=2\alpha_{min}$ and $\delta_{min}= \frac{\delta_{max}}{2}$, simplifies to:

$$\alpha\delta=\alpha_{min}\delta_{max}\left( 1+e^{-\theta_{s}\rho}-\frac{e^{-\theta_{c}\rho}}{2}-\frac{e^{-\theta_{c}\rho}e^{-s\rho}}{2} \right).$$

We note that our estimated, overall baseline transmission rate ($\bar{\beta}$) $= \alpha_{min}\delta_{max}$, resulting in:

$$\alpha\delta=\bar{\beta}\left( 1+e^{-\theta_{s}\rho}-\frac{e^{-\theta_{c}\rho}}{2}-\frac{e^{-\theta_{c}\rho}e^{-s\rho}}{2} \right).$$

Hence the net transmission rate in all models depends purely on the observed (estimated) baseline net transmission rate $\bar{\beta}$, and on fixed combinations of the two sensitivity parameters, $\theta_{c}$ and $\theta_{s}$, and the assumed provisioning rate $\rho$; it does not depend on how we specifically partition that transmission rate into the contact and susceptibility components (i.e., the relative magnitudes of $\alpha_{min}$ and $\delta_{max}$, providing their product equals $\bar{\beta}$). For simplicity therefore, we arbitrarily assume the baseline susceptibility probability ($\delta_{max}$) is a value in the range 0.05 – 1 (reflecting a relatively high probability of infection given contact), and the baseline contact rate ($\alpha_{min}$) is a negative power of 10 (i.e., 10^-1^; 10^-2^ etc), such that $\alpha_{min}\delta_{max}= \bar{\beta}$. For example, for the nematode *H. polygyrus*, the estimated baseline transmission rate is $\bar{\beta}= 2.05x{10}^{-4}$ (see below), from which we assume $\alpha_{min}$ = 0.001 and $\delta_{max}$ = 0.205. Again, we emphasise our results are not sensitive to alternative partitioning of these parameters.

Finally, we note that since cowpox infections were so rare in our system (<5%), we estimated its recovery rate and prevalence in wood mice from Telfer et al. (2002). They reported infections lasting ~1 month; hence recovery rate was $0.25\frac{1}{ind \cdot week}$ They also reported seroprevalence in wood mice ranging $0\%-17\%$, so we assumed a mean of $0.085$. As described above, since animals can retain antibodies after clearing infections, seroprevalence reflects both animals with on-going infections, and those that have recovered: (*I+R*)/*N*. By this definition, the equilibrium seroprevalence from our SIR model is $\frac{N\alpha\delta-(\gamma+\mu)}{N\alpha\delta}$ which, given the parameter estimates from our demographic model fitting, gives an estimated transmission rate of $0.009\frac{1}{ind \cdot week}$.

**Parameter estimation results**

Host birth rate was estimated to be $1.24 [1.23-1.25]$ $\frac{ind}{ind \cdot week}$and mortality rate was $\frac{1}{13.75 [13.57-13.94] weeks}$. The estimated carrying capacity was $42.12 [42.05-42.19]$ mice per grid. All estimated disease-related parameters are shown in Table S1.

| Parasite | Parameter | Parameter Estimate | 95% CI |
| --- | --- | --- | --- |
| *Heligmosomoides polygyrus* (macroparasite) | Transmission rate ($\alpha\delta$)  Disease-induced mortality ($\nu$) | 2.05x10^-4^  0.765 | 2.04x10^-4^ – 2.06x01^-4^  0.763 – 0.767 |
| Cowpox virus (SIR) | Transmission rate ($\alpha\delta$)  Recovery rate ($\gamma$) | 0.009  0.25 | * |
| *Eimeria hungaryensis* (SIS) | Transmission rate ($\alpha\delta$)  Recovery rate ($\gamma$) | 9.59x10^-7^  0.1131 | 9.58x10^-7^ – 9.61x01^-7^  0.1128 – 0.1134 |
| Herpesvirus (SAL) | M🡪M Transmission ($\alpha_{1}\delta_{1}$)  F🡪M Transmission ($\alpha_{2}\delta_{2}$)  M🡪F Transmission ($\alpha_{3}\delta_{3}$)  F🡪F Transmission ($\alpha_{4}\delta_{4}$)  Acute to latent transition ($\varepsilon$)  Latent to acute transition ($\eta$) | 0.0255  0.0424  0.0148  0.0156  0.815  0.0132 | 0.0253 – 0.0256  0.0421 – 0.0428  0.0147 – 0.0149  0.0154 – 0.0157  0.813 – 0.817  0.0131 – 0.0133 |
| *Bartonella* spp. (vector-borne) | H🡪V Transmission ($\alpha\omega$)  H🡪V Transmission ($\alpha\delta$)  Recovery rate ($\gamma$) | 0.506  8.15x10^-3^  0.643 | 0.503 – 0.509  8.11x10^-3^ – 8.18x10^-3^  0.639 – 0.656 |
| *Trypanosoma grosi* (vector-borne) | H🡪V Transmission ($\alpha\omega$)  H🡪V Transmission ($\alpha\delta$)  Recovery rate ($\gamma$) | 0.547  1.056x10^-3^  0.644 | 0.544 – 0.551  1.052x10^-3^ – 1.061x10^-3^  0.641 – 0.647 |

**Table S1. Parasite-related parameter estimates.** Values show median and 95% credible intervals from the posterior distributions of each parameter. *Since cowpox infections were very rare in our dataset we estimated parameter from Telfer et al., ( 2002), and were not able estimate 95% CIs for parameter estimates.

**
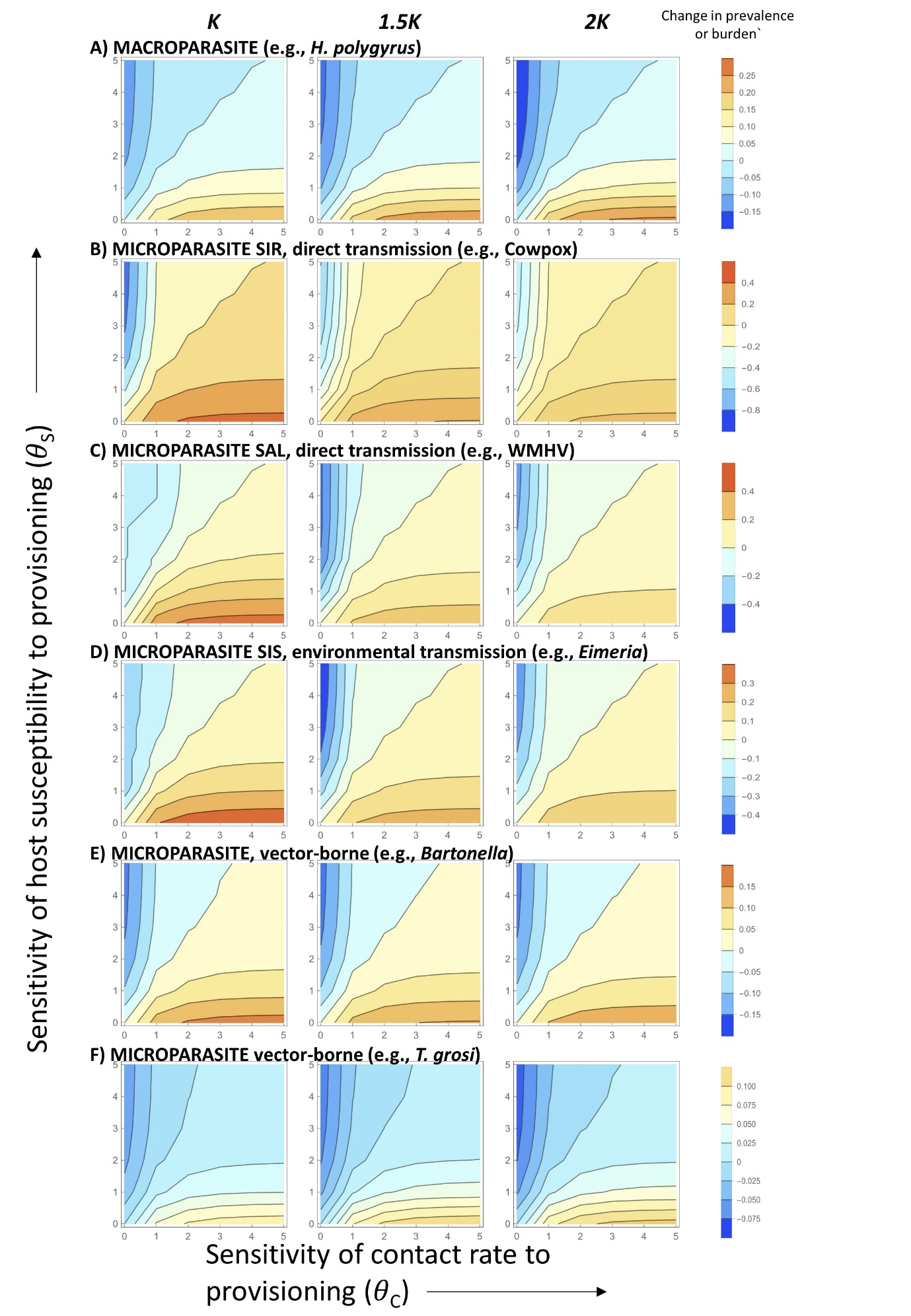
**

**Figure S1. Resource-provision impacts parasite infection and burdens.** As in Figure 3 in the main text, the colours show the magnitude of those differences; increasing redness shows provisioning has an increasingly positive effect on that parasite’s burden or prevalence, increasing blueness shows provisioning has an increasingly negatively effect on burden or prevalence. Row A shows macroparasite mean burden change and B row to F row represent microparasites’ prevalence changes (the difference in equilibrium prevalence between provisioned ($\rho=1$) and the un-provisioned baseline ($\rho=0$) scenarios). For all figures, the *X-*axis represents the sensitivity of contact rate to provisioning $(\theta_{c})$ and *y-*axis is the sensitivity of host susceptibility to provisioning $\left( \theta_{s} \right).$Columns illustrate different carrying capacities, varying from $K$ to $2K$, representing increasing levels of host demographic response to provisioning. Note, this figure is equivalent to Figure 3 in the main text, except the colour-coding scale here is consistent across panels (columns) for each parasite species, but vary between parasite species (rows).


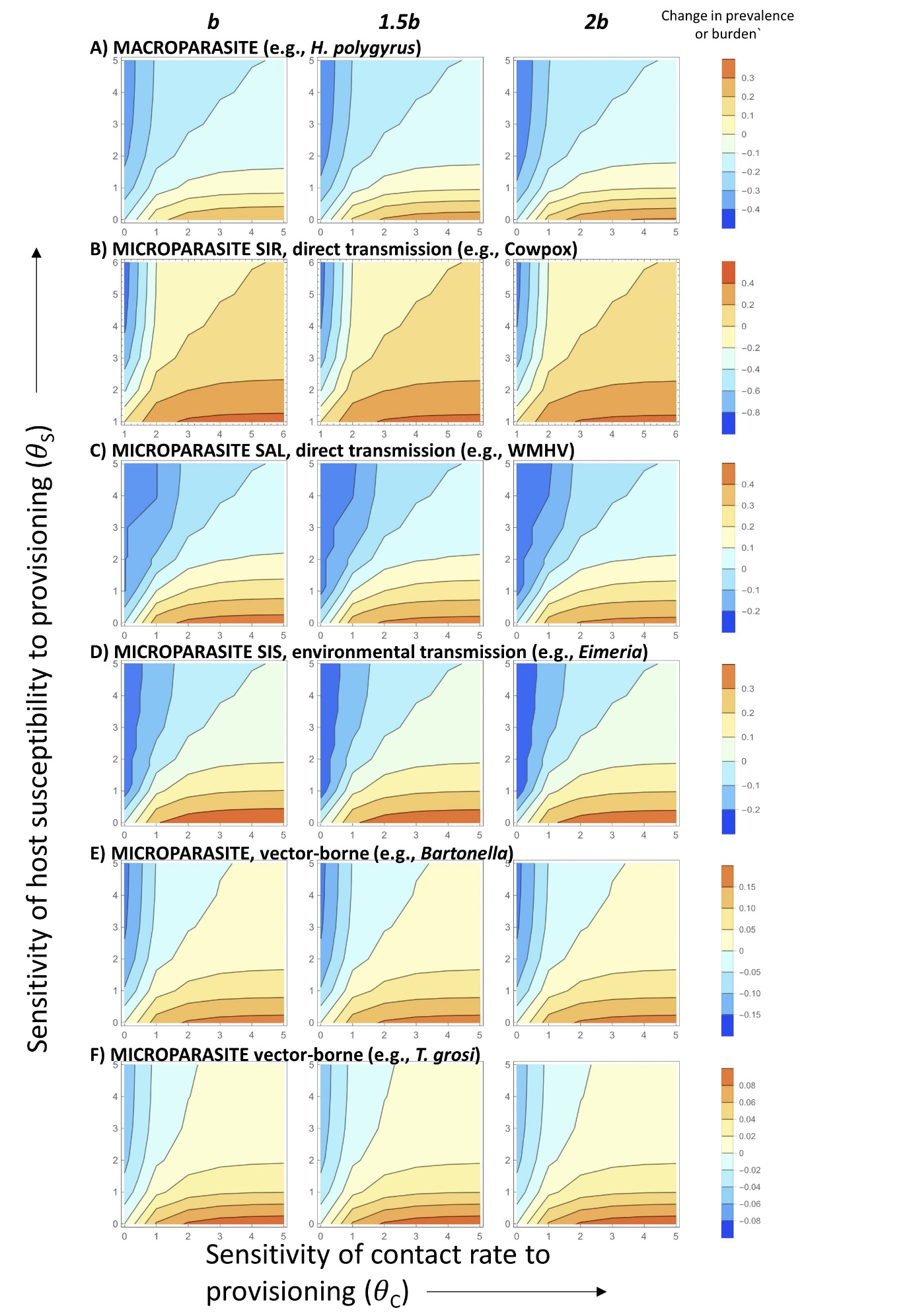


**Figure S2. Parasite outcome changes induced by provisioning, whereby considering demographic changes due to provisioning that affect host birth rate (*b*).** Row A shows macroparasite mean burden change and B row to F row represent microparasites’ prevalence changes (the difference in equilibrium prevalence between provisioned ($\rho=1$) and the un-provisioned baseline ($\rho=0$) scenarios). The colours show the magnitude of those differences; increasing redness shows provisioning has an increasingly positive effect on that parasite’s burden or prevalence, increasing blueness shows provisioning has an increasingly negatively effect on burden or prevalence. For all figures, the *X-*axis represents the sensitivity of contact rate to provisioning $(\theta_{c})$ and *y-*axis is the sensitivity of host susceptibility to provisioning $\left( \theta_{s} \right).$Columns illustrate scenarios varying from $b$ (left-hand column) to $2b$ (right-hand column).


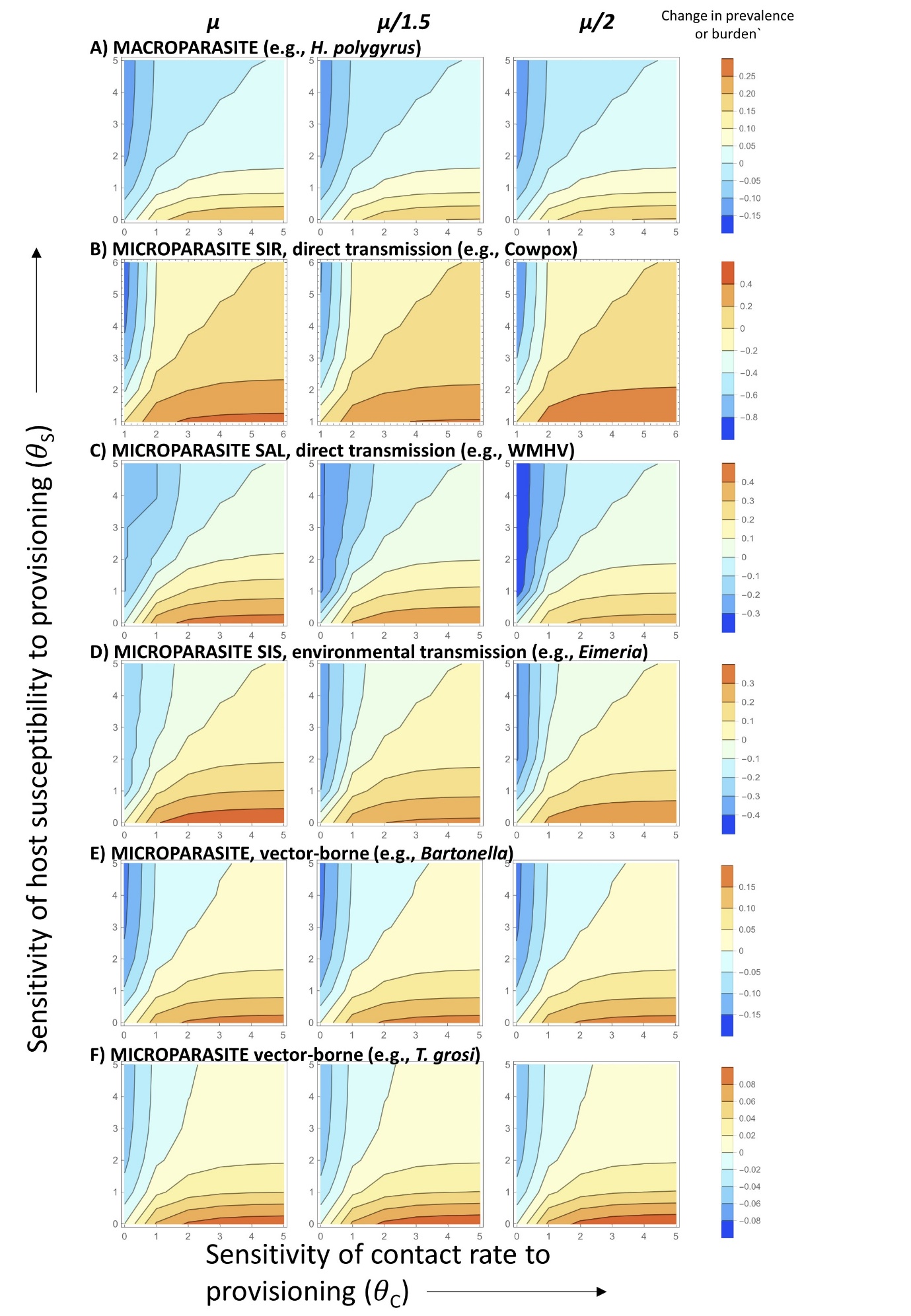


**Figure S3. Parasite outcome changes induced by provisioning, whereby demographic changes due to provisioning that affect host death rate (μ)**. Row A shows macroparasite mean burden change and B row to F row represent microparasites’ prevalence changes (the difference in equilibrium prevalence between provisioned ($\rho=1$) and the un-provisioned baseline ($\rho=0$) scenarios). The colours show the magnitude of those differences; increasing redness shows provisioning has an increasingly positive effect on that parasite’s burden or prevalence, increasing blueness shows provisioning has an increasingly negatively effect on burden or prevalence. For all figures, the *X-*axis represents the sensitivity of contact rate to provisioning $(\theta_{c})$ and *y-*axis is the sensitivity of host susceptibility to provisioning $\left( \theta_{s} \right).$Columns illustrate scenarios varying from *μ* (left-hand column) to $\mu/2$ (right-hand column).

**References**

Anderson, R. M., & May, R. M. (1978). Regulation and stability of hostparasite Anim., population interactions: I. Regulatory processes. *Journal of Animal Ecology*, *47*, 219–247.

Camacho, A., & Funk, S. (2019). *fitR Tool box for fitting dynamic infectious disease models to time series*.

Erazo, D., Pedersen, A. B., Gallagher, K., & Fenton, A. (2021). Who acquires infection from whom? Estimating herpesvirus transmission rates between wild rodent host groups. *Epidemics*. doi: 10.1016/j.epidem.2021.100451

Knowles, S. C. L., Fenton, A., & Pedersen, A. B. (2012). Epidemiology and fitness effects of wood mouse herpesvirus in a natural host population. *Journal of General Virology*, *93*(PART 11), 2447–2456. doi: 10.1099/vir.0.044826-0

Knowles, S. C. L., Fenton, A., Petchey, O. L., Jones, T. R., Barber, R., & Pedersen, A. B. (2013). Stability of within-host-parasite communities in a wild mammal system. *Proceedings of the Royal Society B: Biological Sciences*, *280*(1762). doi: 10.1098/rspb.2013.0598

Smith, M. J., Telfer, S., Kallio, E. R., Burthe, S., Cook, A. R., Lambin, X., & Begon, M. (2009). Host-pathogen time series data in wildlife support a transmission function between density and frequency dependence. *Proceedings of the National Academy of Sciences of the United States of America*. doi: 10.1073/pnas.0809145106

Sweeny, A. R., Albery, G. F., Venkatesan, S., Fenton, A., & Pedersen, A. B. (2021). Spatiotemporal variation in drivers of parasitism in a wild wood mouse population. *Functional Ecology*. doi: 10.1111/1365-2435.13786

Telfer, S., Bennett, M., Bown, K., Cavanagh, R., Crespin, L., Hazel, S., … Begon, M. (2002). The effects of cowpox virus on survival in natural rodent populations: Increases and decreases. *Journal of Animal Ecology*. doi: 10.1046/j.1365-2656.2002.00623.x

Withenshaw, S. M., Devevey, G., Pedersen, A. B., & Fenton, A. (2016). Multihost Bartonella parasites display covert host specificity even when transmitted by generalist vectors. *Journal of Animal Ecology*. doi: 10.1111/1365-2656.12568
